# Limb-salvage surgery using personalized 3D-printed porous tantalum prosthesis for distal radial osteosarcoma: A case report

**DOI:** 10.1097/MD.0000000000027899

**Published:** 2021-11-19

**Authors:** Ge Chen, Yiran Yin, Chang Chen

**Affiliations:** a Department of Orthopedics, Affiliated Hospital of Southwest Medical University, Luzhou City, Sichuan Province, China; b Department of Orthopedics, Ziyang First People‘s Hospital, Ziyang, Sichuan Province, China.

**Keywords:** bone defect, case report, customized implants, osteosarcoma, tantalum, three-dimensional printing, wrist reconstruction

## Abstract

**Rationale:**

Three-dimensional (3D) printing has been widely utilized for treating the tumors of bone and soft tissue. We herewith report a unique case of distal radial osteosarcoma who was treated with a 3D printed porous tantalum prosthesis.

Patient concerns: A 58-year-old Chinese male patient presented to our clinic complaining about a 6-month history of a progressive pain at his right hand, associated with a growing lump 2 months later.

**Diagnosis:**

Osteosarcoma of distal radius confirmed by percutaneous biopsy and tumor biopsy.

**Interventions:**

A limb-salvage surgery was performed with a 3D printed porous tantalum prosthesis, combined with the postoperative chemotherapy for 4 cycles.

**Outcomes:**

At 2-year follow-up, complete pain relief and satisfactory functional recovery of his right wrist were observed.

**Lessons:**

Personalized 3D printed prosthesis is an effective and feasible method for treating the osteosarcoma and reconstruction of complex bone defects.

## 1 Introduction

Although osteosarcoma is the most frequent primary malignant bone tumor characterized by high metastasis and recurrence rates and low survival rates,^[1]^ <1% of osteosarcoma occurs in the distal radius.^[2]^ Once an osteosarcoma was diagnosed, surgery and postoperative chemotherapy were recommended for long-term survival.^[3]^ For wrist osteosarcoma, amputation should be avoided because it might result in severe physical disability and psychosocial trauma.^[4]^ Limb-salvage surgery is the golden standard for restoring function unless there were massive neurovascular invasion.^[5]^ As completed resection is essential for preventing the tumor recurrence,^[6]^ an effective reconstruction of limb and joint is imperative and difficult. Because the hand and the wrist are complex and have a more refined function than any other part of the body, large tumor resection-induced bone defects might result in the abnormal appearance, shortening of limb, and poor postoperative function.^[7,8]^ How to restore the wrist function as satisfactory as possible through the surgical techniques is worthy of researching. Personalized 3-dimensional (3D) printed prosthesis could be an ideal option for restoring the functions after the limb-salvage surgery of osteosarcoma.^[9]^

Here we reported a case of distal radial osteosarcoma who was treated with a 3D printed porous tantalum prosthesis.

## 2 Case presentation

A 58-year-old Chinese male patient presented to our clinic complaining about a 6-month history of a progressive pain at his right hand, associated with a growing lump 2 months later. Limited range of motion (ROM) of the right wrist was reported as well. Physical examination indicated the lump was an approximately 5 cm × 3 cm hard mass on the radial side of the right wrist, with obvious tenderness and unclear boundary. The skin was slightly red and swollen without ulceration. The right wrist was unable to flexion or extension due to the severe pain, with flexing limitation of his fingers.

**Figure 1:**
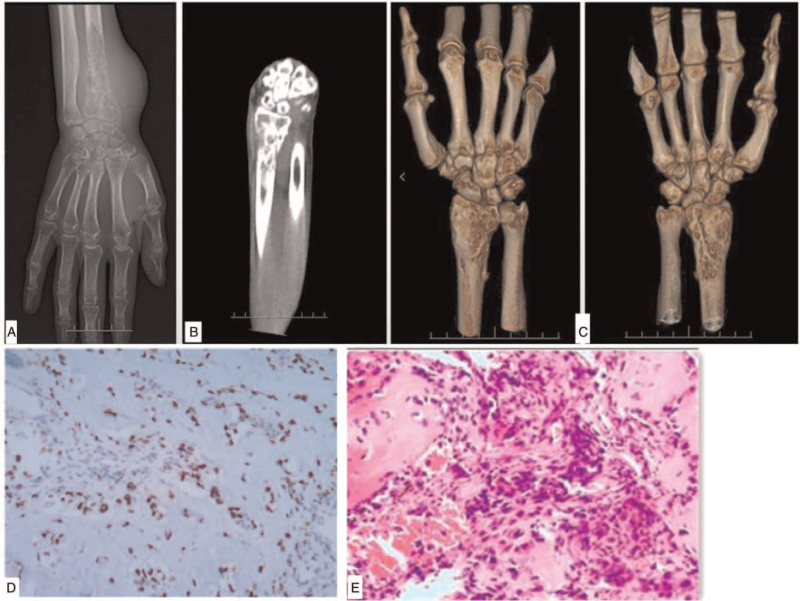
Preoperative examination. (A–C) The x-ray film and computed tomography scan. multiple osteolytic bone destruction occurred at the distal end of the right radius and the surface of wrist joint, with several discontinuous bone cortex. The distal bone was slightly enlarged, with slight periosteal reaction. d.e pathological biopsy. (D) Preoperative biology and (E) second biology at the surgery: osteogenic malignant tumor.

The x-ray film and computed tomography (CT) scan showed multiple osteolytic bone destruction occurred at the distal end of the right radius and the surface of wrist joint, with several discontinuous bone cortex. The distal bone was slightly enlarged, with slight periosteal reaction. After admitted to the ward, a percutaneous bone biopsy was performed, revealing osteogenic malignant tumor (Fig. 1). A diagnosis of osteosarcoma was suggested.

Considering the rapid progress of tumor, after the patient and his family members gave informed consent (Informed consent was obtained from the patient for publication of this case report details), we decided to perform a limb-salvage procedure to remove the tumor and reconstruct the wrist joint. In order to reconstruct the large structural defect after removal of the tumor without compromising the length and normal appearance of the radius, using a personalized 3D printed prosthesis was considered to be an ideal option.

**Figure 2:**
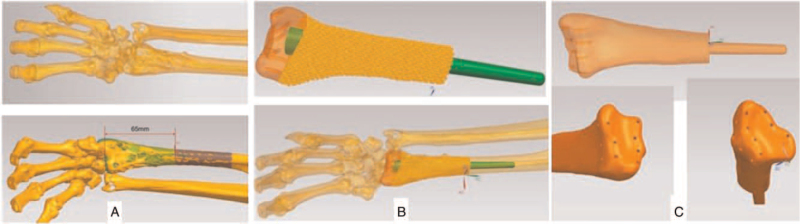
3D remodeling and prosthesis designing. (A) The osteotomy boundary was accurately located 5 mm proximal to the tumor boundary. (B) The length of the prosthetic intramedullary rod was set to 40 mm to ensure sufficient stability. (C) Suture holes on wrist joint surface provided effective attachment points for soft tissues and muscles.

The CT scan of hand, wrist and forearm were obtained by a scanner (Siemens) with 1.0 mm each layer. The data were stored and analyzed by Mimics 17.0 software in digital imaging and communications in medicine (DICOM) format. After the right radius and the wrist were remodeled by the Mimics 17.0 software (Mimics, Materialise, Leuven, Belguim), it was imported into SIEMENS NX software (Siemens PLM Software Inc., Germany) to design the guiding plate. The design of guiding plate was restored in stereolithography (STL) format and then printed by UP BOX+ 3D printer (Beijing Tiertime Technology Co., Ltd, China) using polylactic acid (PLA). 3D printing remodel based on the healthy contralateral left limb was conducted to simulate the prosthesis of right wrist. In preoperative plan, the customized guide plate should accurately locate the osteotomy boundary 5 mm beyond the tumor boundary to ensure a safe resection limit and reduce the operation time. The stem of prosthesis was set to 40 mm so that enough stability in the medullary cavity of radius can be ensured. Meanwhile, a longer stem can effectively prevent the rotation of prosthesis. The suture holes on the surface of wrist joint were designed to provide attachments for soft tissue and muscles, increasing the stability of prosthesis and attaining a better function (Fig. 2).

**Figure 3:**
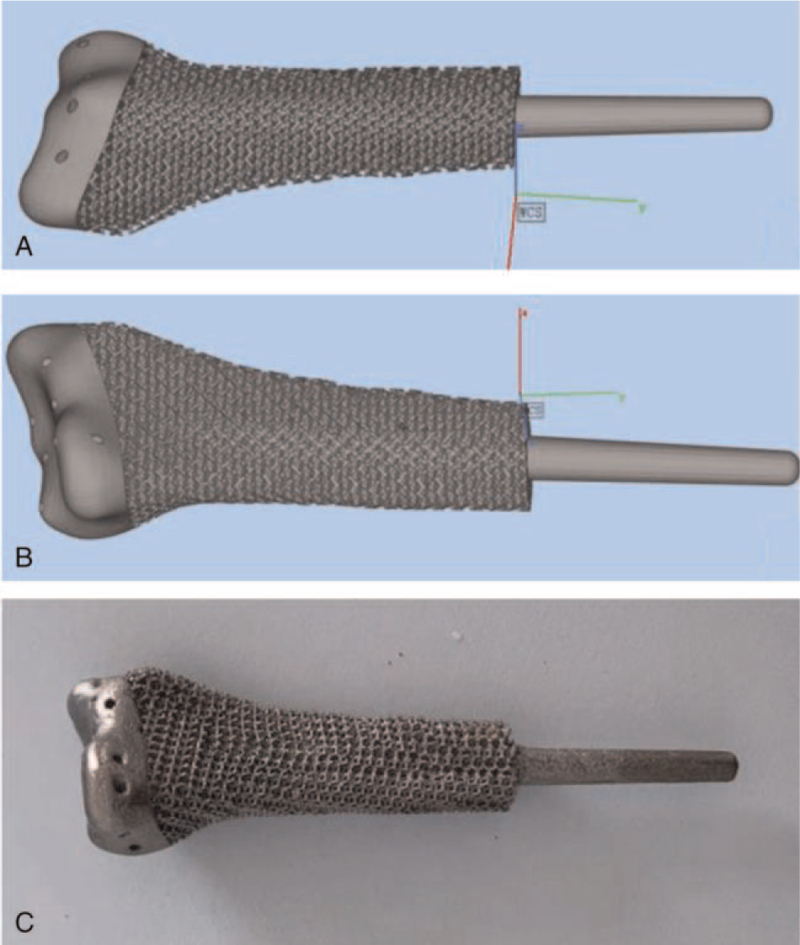
The prosthesis used in the current case. (A and B) Final design. (C) Customized prosthesis.

**Figure 4:**
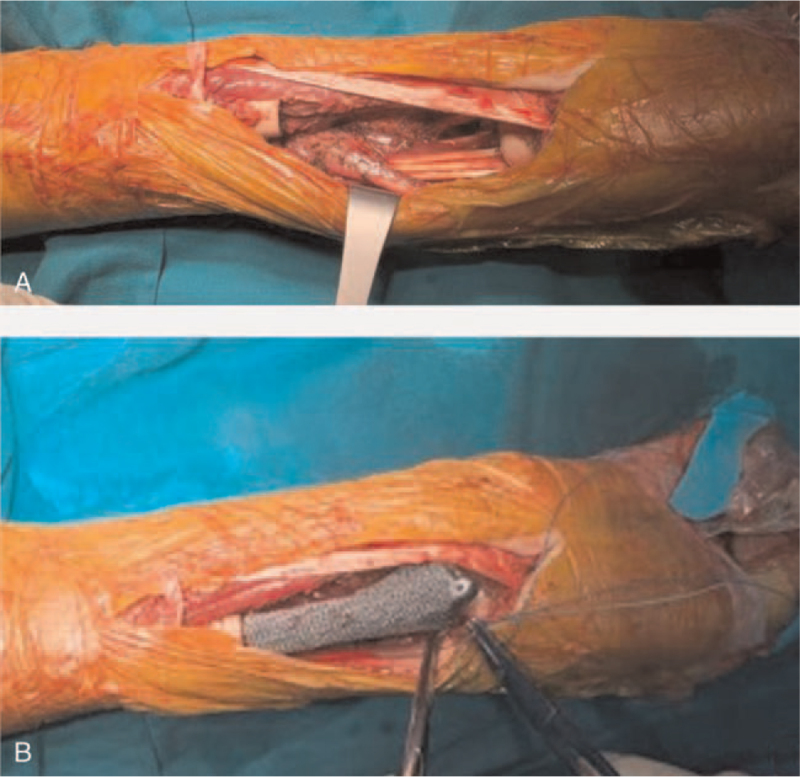
The surgical procedure. (A) Tumor resection and (B) 3D printing prosthesis implantation.

The surgical procedure was carried out under general anesthesia, with the patient positioned in a supine position. After an adequate exposure of the whole tumor, the invaded flexor carpi radialis tendon and the radial artery were cut and removed. After protecting the peripheral blood vessels and nerves, the ulna was dissociated with the repeated cauterization of the surface using the electric knife. After removed the invaded joint capsule and exposing the wrist joint, the distal radius was successfully isolated. Guided by at the 3D printed osteotomy plate, the tumor on the radius was removed and sent for pathological biopsy (Fig. 4). After reconfirming that the resected margins was free of tumor cells (Fig. 4: the proximal radius stump was normal bone tissue), the proximal medullary cavity was scraped off with a curette and cauterized with an electric knife. After washed repeated by sterile water, the proximal medullary cavity was carefully reamed. Finally, the prosthesis (Fig. 3) was implanted, and further stability was ensured by wire binding on the radius. The wrist joint capsule and tendons were sutured to the distal end of the prosthesis. The operation was finished within 54 minutes.

**Figure 5:**
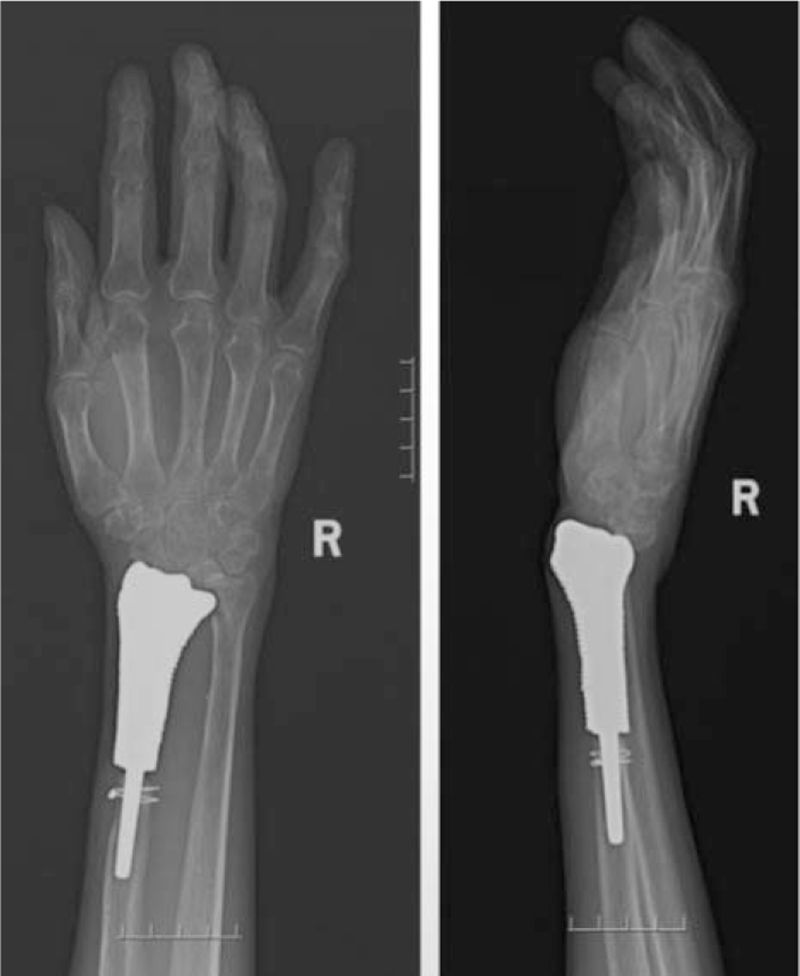
X-ray films 2 year after operation: There was no failure or loosening of implant.

The patient achieved a fast recovery after the surgical procedure. The forearm and wrist were fixed with plaster cast for 4 weeks. The wound drainage tube was removed on the third day after operation. Antibiotics were taken orally for 2 weeks postoperatively. The staples were removed on POD 14, and the wound healed well. Postoperative pathological biospy suggested an osteoplastic tumor. The immunohistochemical results showed that SATB2 (+), Ki67 (+, 40%), S100 (−), P63 (−), P53 (−), supporting the diagnosis of angiodilated osteosarcoma. Sixteen days after operation, the patient received postoperative adjuvant chemotherapy for 4 cycles, with the typical AP scheme: doxorubicin (25 mg/m^2^) 40 mg; d1-3+ cisplatin (25 mg/m^2^) 40 mg; adriamycin cumulative dose 75 mg. Until the end of chemotherapy, the patient's blood examinations were not significantly abnormal. Four weeks after the operation, the rehabilitation was started after the plaster cast was removed. In one-year follow-up, the patient had satisfactory extension and limited flexion of right hand. The range of motion of the radiocarpal joint and forearm is as follows: straight: 25 °, lexion: 20 °, pronation: 25 °, Supination: 15 °. Slight pain would occur in the right wrist occasionally. X-ray and magnetic resonance imaging showed no local recurrence and slight bone resorption between prosthesis and radius 24 months after operation (Fig. 5). Chest CT showed no lung metastasis at the latest follow-up.

## 3 Discussion and conclusion

Osteosarcoma of distal radius was rarely found in clinical practice, accounting for less than 1% of the overall osteosarcoma.^[10]^ For wrist osteosarcoma, amputation can not only leads to physical disability, but also may have profound social and psychological effects.^[11,12]^ Current studies suggested that osteosarcomas of the hand and wrist were less likely to be high-grade, ensuring a good long-term prognosis after limb-salvage surgery.^[13]^ In our case, the tumor was large and grew rapidly, which would cause a huge bone defect after resection. A personalized reconstruction of the wrist and radius was essential for obtaining long-term satisfactory function postoperatively. 3D printing technology has been widely utilized in medical field due to its rapid remodeling and precise customization.^[14]^ The indications of reconstruction using 3D printing technology in tumor related bone defects remained controversial. Previously, autografts were most commonly used for various bone substitution, characterized with obvious limitations including poor availability, donor-site morbidity, and prolonged operation time.^[15]^ When critical-sized and irregular-shaped bone defects resulted from bone tumor resection or high-energy injuries, either autografts or regular prosthesis can achieve a satisfactory anatomic reconstruction.^[16]^ Meanwhile, malignant bone tumors had high incidence rate in adolescences and younger adults, requiring a better long-term function restoration. Those situations could be ideal indications for personalized prosthesis fabricated by 3D printing technology. Besides customizing highly matched prosthesis, 3D printing can also fabricate bio-scaffolds as a universal therapeutic platform for synergistic therapy of osteosarcoma.^[14]^ It had several advantages in our case, including determining the resection boundary of tumor, simulating the operation process, and customizing the personalized 3D printing prosthesis, which can effectively avoid the prosthesis mismatch and obtain more beautiful postoperative appearance. Meanwhile, the design of suture holes on the surface of the prosthesis provides a solid reconstruction for the muscles and soft tissue, ensuring an early functional exercise and a better long-term wrist and hand function. The intramedullary rod was designed for the fixation between the prosthesis and the proximal radius. Compared with the cement fixation, porous tantalum coating can ensure better early bone growth, reducing the risk of bone dissolution and prosthesis loosening.^[17]^

The 3D printed guiding plate can accurately ensure the tumor boundary and osteotomy range in the operation,^[18]^ preserving a sufficient length of the proximal radius. In our case, satisfactory symmetry and limb length was obtained through the customized prosthesis according to the contralateral limb. Our patient reported a significant pain relief associated with a functional improvement at 2-year follow-up. A case report by Higuchi et al^[4]^ demonstrated a limb-salvage surgery with en bloc tumor excision and reconstruction using frozen autografts, which showed satisfactory results nearly 4 years postoperatively in terms of the function of the affected limb. They suggested that recycled bone, including frozen autografts, can perfectly reconstruct the original site and can reproduce the proper anatomical alignment. However, compared with their case, our patient showed a broken joint surface and enlarged distal radius, making autografts not suitable for our reconstruction. Hatano et al^[19]^ reported a 9-year-old girl with osteosarcoma of the radius treated with segmental forearm resection and replantation followed by forearm lengthening of 11 cm. At 9-year follow-up, the patient had recovered sensory function, and her pinch and grasp were sufficient for performing daily activities.

In this case, we chose the porous tantalum as the material of tumor prosthesis due to its unique biomechanical properties. Because of its preeminent corrosion resistance, toughness, and bioactivity, tantalum has been widely used as implant material in various medical fields. The study of Fan et al showed that compared with titanium, tantalum has lower range of Young's modulus, which could effectively reduce the effect of stress shielding.^[20]^ Meanwhile, the porous tantalum are strong enough to be used as implants, for reconstructing the large bone defect. The compression strength and bearing strength of porous tantalum can be further increased when osseous integration completed 4 to 6 weeks after surgery, with a higher long-term osteoid formation compared with titanium. The study of Lu et al^[21]^ used bone marrow mesenchymal stem cells from ovariectomized rats found that porous tantalum had a better cell adhesion, proliferation, and osteogenic differentiation than titanium plates. Meanwhile, the use of 3D printing technologies made it convenient for us to control the shape and pore parameters of our scaffold, ensuring a better biocompatibility and match of this patient. However, in our experience, even though there were many excellent biomechanical properties of the porous tantalum, it was still not suitable for every bone defect reconstruction. If bone defects existed in facial bone,^[22]^ clavicle^[23]^ or chest wall,^[24]^ the materials with lower density and smoother surface like polyether ketone could be more suitable for the reconstruction than tantalum.

As a new trend for customizing personalized prosthesis,^[25]^ the application of 3D printing technology in China was rudimentary and needs more research. We shared our cases and experience so as to provide references to treat bone tumor with this technology. First, remodeling and designing are the most important steps for the prosthesis, requiring professional teams and specific equipment to ensure that the size of the prosthesis could precisely match the site of bone defect. Based on previous bone tumor cases treated with 3D printing in our institution,^[18,23,26]^ the process of designing and printing was effectively shortened. However, most medical institutions did not have the condition and experience, which might limit the application and development of the 3D printing technology for treating bone tumor. Meanwhile, as raw material having better bone ingrowth and biomechanical properties, tantalum has high density and high cost. How to design appropriate porosity and prosthesis shape under the condition of reducing weight and saving cost still needs research. Meanwhile, the traditional materials like titanium and tantalum metal prostheses might have high density and are easy to produce stress shielding,^[27]^ which may lead to long-term complications including prosthesis loosening, periprosthetic osteoporosis and osteolysis.^[28]^ As there were so many biomaterials which can be chosen for bone defect reconstruction, how to choose the most suitable prosthesis material according to various types of bone defects is an important subject that needs further research. Still, as the advantages of 3D printing far outweigh its disadvantages, we do believe that 3D-printed implants will be widely used in the future and will benefit millions of patients.

## 4 Conclusions

This study suggested that personalized 3D printed prosthesis is an effective and feasible method for treating the osteosarcoma and reconstruction of complex bone defects. We also suggested that the advantages and complications of osteosarcomatous reconstruction should be carefully discussed due to limited evidence of this pretty new technology. Meanwhile, in our case, the clinical outcomes of surgical reconstruction for treating distal radial osteosarcoma were satisfactory in functional aspect. However, it remains an isolated case and further reports are awaited to help surgeons and patients in their decision process.

## Author contributions

**Conceptualization:** Ge Chen, Yiran Yin, Chang Chen.

**Data curation:** Ge Chen, Yiran Yin, Chang Chen.

**Formal analysis:** Ge Chen, Yiran Yin, Chang Chen.

**Funding acquisition:** Ge Chen, Chang Chen.

**Investigation:** Chang Chen.

**Methodology:** Chang Chen.

**Project administration:** Chang Chen.

**Resources:** Ge Chen, Chang Chen.

**Software:** Chang Chen.

**Supervision:** Ge Chen, Chang Chen.

**Validation:** Ge Chen, Chang Chen.

**Visualization:** Chang Chen.

**Writing – original draft:** Chang Chen.

**Writing – review & editing:** Chang Chen.

## References

[1] Czarnecka AM Synoradzki K Firlej W. Molecular biology of osteosarcoma. Cancers (Basel) 2020;12:10.3390/cancers12082130 PMC7463657 32751922

[2] Pradhan A Reddy KI Grimer RJ. Osteosarcomas in the upper distal extremities: are their oncological outcomes similar to other sites? Eur J Surg Oncol 2015;41:407–12. 25442503 10.1016/j.ejso.2014.11.038

[3] Gill J Gorlick R. Advancing therapy for osteosarcoma. Nat Rev Clin Oncol 2021. 10.1038/s41571-021-00519-8 34131316

[4] Higuchi T Yamamoto N Hayashi K. Successful joint preservation of distal radius osteosarcoma by en bloc tumor excision and reconstruction using a tumor bearing frozen autograft: a case report. BMC Surg 2018;18:12. 29490656 10.1186/s12893-018-0346-y PMC5831224

[5] Qi L Ren X Liu Z. Predictors and survival of patients with osteosarcoma after limb salvage versus amputation: a population-based analysis with propensity score matching. World J Surg 2020;44:2201–10. 32170370 10.1007/s00268-020-05471-9

[6] Traven SA Brinton DL Walton ZJ. A propensity-score matched analysis of limb salvage vs amputation for osteosarcoma. J Surg Oncol 2019;120:1252–8. 31486107 10.1002/jso.25701

[7] Grinberg SZ Posta A Weber KL. Limb salvage and reconstruction options in osteosarcoma. Adv Exp Med Biol 2020;1257():13–29. 32483727 10.1007/978-3-030-43032-0_2

[8] Agarwal M Anchan C Shah M. Limb salvage surgery for osteosarcoma: effective low-cost treatment. Clin Orthop Relat Res 2007;459:82–91. 17417098 10.1097/BLO.0b013e31805d85c4

[9] Jovičić MŠ Vuletić F Ribičić T Šimunić S Petrović T Kolundžić R. Implementation of the three-dimensional printing technology in treatment of bone tumours: a case series. Int Orthop 2021;45:1079–85. 32901331 10.1007/s00264-020-04787-4

[10] Kelley LM Schlegel M Hecker-Nolting S. Pathological fracture and prognosis of high-grade osteosarcoma of the extremities: an analysis of 2,847 Consecutive Cooperative Osteosarcoma Study Group (COSS) patients. J Clin Oncol 2020;38:823–33. 31928458 10.1200/JCO.19.00827

[11] Ottaviani G Robert RS Huh WW. Sociooccupational and physical outcomes more than 20 years after the diagnosis of osteosarcoma in children and adolescents: limb salvage versus amputation. Cancer 2013;119:3727–36. 23907996 10.1002/cncr.28277 PMC3842284

[12] Agarwal M. CORR insights?: Is limb salvage with microwave-induced hyperthermia better than amputation for osteosarcoma of the distal tibia? Clin Orthop Relat Res 2017;475:1678–80. 28265886 10.1007/s11999-017-5305-x PMC5406351

[13] Huang Q Liang X Ren T. The role of tumor-associated macrophages in osteosarcoma progression - therapeutic implications. Cell Oncol (Dordr) 2021;44:525–39. 33788151 10.1007/s13402-021-00598-w PMC12980758

[14] Dang W Yi K Ju E. 3D printed bioceramic scaffolds as a universal therapeutic platform for synergistic therapy of osteosarcoma. ACS Appl Mater Interfaces 2021;13:18488–99. 33856761 10.1021/acsami.1c00553

[15] Zhang L Yang G Johnson BN Jia X. Three-dimensional (3D) printed scaffold and material selection for bone repair. Acta Biomater 2019;84:16–33. 30481607 10.1016/j.actbio.2018.11.039

[16] Nulty J Freeman FE Browe DC. 3D bioprinting of prevascularised implants for the repair of critically-sized bone defects. Acta Biomater 2021;126:154–69. 33705989 10.1016/j.actbio.2021.03.003

[17] Bandyopadhyay A Mitra I Shivaram A. Direct comparison of additively manufactured porous titanium and tantalum implants towards in vivo osseointegration. Addit Manuf 2019;28:259–66. 31406683 10.1016/j.addma.2019.04.025 PMC6690615

[18] Chen G Muheremu A Yang L. Three-dimensional printed implant for reconstruction of pelvic bone after removal of giant chondrosarcoma: a case report. J Int Med Res 2020;48:300060520917275. 32290744 10.1177/0300060520917275 PMC7160782

[19] Hatano H Morita T Kobayashi H. Osteosarcoma of the distal radius treated with segmental forearm resection, hand replantation, and subsequent limb lengthening: case report. J Hand Surg Am 2014;39:1155–9. 24810936 10.1016/j.jhsa.2014.03.030

[20] Fan H Deng S Tang W. Highly porous 3D printed tantalum scaffolds have better biomechanical and microstructural properties than titanium scaffolds. Biomed Res Int 2021;2021:2899043 Published 2021 Sep 28. 34621893 10.1155/2021/2899043 PMC8492259

[21] Lu MM Wu PS Guo XJ Yin LL Cao HL Zou D. Osteoinductive effects of tantalum and titanium on bone mesenchymal stromal cells and bone formation in ovariectomized rats. Eur Rev Med Pharmacol Sci 2018;22:7087–104. 30468450 10.26355/eurrev_201811_16241

[22] Laux CJ Hodel SM Farshad M Müller DA. Carbon fibre/polyether ether ketone (CF/PEEK) implants in orthopaedic oncology. World J Surg Oncol 2018;16:241 Published 2018 Dec 28. 30593277 10.1186/s12957-018-1545-9 PMC6310953

[23] Chen C Yin Y Xu H. Personalized three-dimensional printed polyether-ether-ketone prosthesis for reconstruction after subtotal removal of chronic clavicle osteomyelitis: a case report. Medicine (Baltimore) 2021;100:e25703. 33907152 10.1097/MD.0000000000025703 PMC8083998

[24] Wang L Liu X Jiang T Huang L. Three-dimensional printed polyether-ether-ketone implant for extensive chest wall reconstruction: a case report. Thorac Cancer 2020;11:2709–12. 32677759 10.1111/1759-7714.13560 PMC7471033

[25] Xu L Qin H Tan J. Clinical study of 3D printed personalized prosthesis in the treatment of bone defect after pelvic tumor resection. J Orthop Translat 2021;29:163–9. 34277347 10.1016/j.jot.2021.05.007 PMC8258599

[26] Li Z Chen G Xiang Y. Treatment of massive iliac chondrosarcoma with personalized three-dimensional printed tantalum implant: a case report and literature review. J Int Med Res 2020;48:300060520959508. 33050744 10.1177/0300060520959508 PMC7570804

[27] Al-Tamimi AA. 3D Topology optimization and mesh dependency for redesigning locking compression plates aiming to reduce stress shielding. Int J Bioprint 2021;7:339. 34286146 10.18063/ijb.v7i3.339 PMC8287512

[28] Huang Q Li X Elkhooly TA. The osteogenic, inflammatory and osteo-immunomodulatory performances of biomedical Ti-Ta metal-metal composite with Ca- and Si-containing bioceramic coatings. Colloids Surf B Biointerfaces 2018;169:49–59. 29747030 10.1016/j.colsurfb.2018.05.010

